# Effective Preparation of *Plasmodium vivax* Field Isolates for High-Throughput Whole Genome Sequencing

**DOI:** 10.1371/journal.pone.0053160

**Published:** 2013-01-04

**Authors:** Sarah Auburn, Jutta Marfurt, Gareth Maslen, Susana Campino, Valentin Ruano Rubio, Magnus Manske, Barbara MacHunter, Enny Kenangalem, Rintis Noviyanti, Leily Trianty, Boni Sebayang, Grennady Wirjanata, Kanlaya Sriprawat, Daniel Alcock, Bronwyn MacInnis, Olivo Miotto, Taane G. Clark, Bruce Russell, Nicholas M. Anstey, François Nosten, Dominic P. Kwiatkowski, Ric N. Price

**Affiliations:** 1 Global and Tropical Health Division, Menzies School of Health Research, Charles Darwin University, Darwin, Australia; 2 Wellcome Trust Sanger Institute, Wellcome Trust Genome Campus, Hinxton, United Kingdom; 3 Medical Research Council Centre for Genomics and Global Health, University of Oxford, Oxford, United Kingdom; 4 Timika Malaria Research Programme, Papuan Health and Community Development Foundation, Timika, Papua, Indonesia; 5 Eijkman Institute for Molecular Biology, Jakarta, Indonesia; 6 Shoklo Malaria Research Unit, Mae Sot, Tak Province, Thailand; 7 Mahidol-Oxford Research Unit, Faculty of Tropical Medicine, Mahidol University, Bangkok, Thailand; 8 Faculties of Infectious and Tropical Diseases and Epidemiology and Population Health, London School of Hygiene and Tropical Medicine, London, United Kingdom; 9 Department of Microbiology, Yong Loo Lin School of Medicine, National University Health System, National University of Singapore, Singapore, Singapore; 10 Division of Medicine, Royal Darwin Hospital, Darwin, Australia; 11 Centre for Tropical Medicine, Nuffield Department of Clinical Medicine, University of Oxford, Oxford, United Kingdom; Universidade Federal de Minas Gerais, Brazil

## Abstract

Whole genome sequencing (WGS) of *Plasmodium vivax* is problematic due to the reliance on clinical isolates which are generally low in parasitaemia and sample volume. Furthermore, clinical isolates contain a significant contaminating background of host DNA which confounds efforts to map short read sequence of the target *P. vivax* DNA. Here, we discuss a methodology to significantly improve the success of *P. vivax* WGS on natural (non-adapted) patient isolates. Using 37 patient isolates from Indonesia, Thailand, and travellers, we assessed the application of CF11-based white blood cell filtration alone and in combination with short term *ex vivo* schizont maturation. Although CF11 filtration reduced human DNA contamination in 8 Indonesian isolates tested, additional short-term culture increased the *P. vivax* DNA yield from a median of 0.15 to 6.2 ng µl^−1^ packed red blood cells (pRBCs) (*p* = 0.001) and reduced the human DNA percentage from a median of 33.9% to 6.22% (*p* = 0.008). Furthermore, post-CF11 and culture samples from Thailand gave a median *P. vivax* DNA yield of 2.34 ng µl^−1^ pRBCs, and 2.65% human DNA. In 22 *P. vivax* patient isolates prepared with the 2-step method, we demonstrate high depth (median 654X coverage) and breadth (≥89%) of coverage on the Illumina GAII and HiSeq platforms. In contrast to the A+T-rich *P. falciparum* genome, negligible bias was observed in coverage depth between coding and non-coding regions of the *P. vivax* genome. This uniform coverage will greatly facilitate the detection of SNPs and copy number variants across the genome, enabling unbiased exploration of the natural diversity in *P. vivax* populations.

## Introduction

*Plasmodium vivax* is a major global health burden. An estimated 2.85 billion people live at risk of infection [1]. Rising levels of multidrug resistance and severe disease have refocused efforts to better understand and control vivax malaria [2]–[8]. One factor hindering investigations into the biology of *P. vivax* has been the inability to maintain this species in continuous culture and subsequent reliance on expensive primate models. Although primate adapted strains of *P. vivax* such as Salvador-1 (Sal-1) have greatly facilitated molecular studies of the parasite, especially in the production of the first WGS for this species [9], [10], it is important that future studies focus on the WGS of *P. vivax* strains currently affecting human populations [11]. Certainly high-throughput sequencing technologies [12]–[14] now provide exciting opportunities to undertake affordable, high-depth whole-genome sequencing of hundreds of *Plasmodium* parasites. The capacity of this approach has been demonstrated in *Plasmodium falciparum*, with whole genome sequencing on 227 *P. falciparum* parasites from across the globe [15]. A similar approach for *P. vivax* is critical to provide insights into the molecular mechanisms underlying drug resistance, disease severity, and the development of the dormant (hypnozoite) liver stage.

However, sequencing *Plasmodium* parasites using high-throughput, shotgun-based methodologies are highly challenging. In addition to the challenges of mapping short-read sequence data and achieving uniform coverage across the genome, low parasitaemias and an abundance of “contaminating” human DNA present critical technical challenges to sample library preparation and data yield. The technical challenges are even greater for *P. vivax* than for *P. falciparum*, owing to restricted *in vitro* culture and log order lower *P. vivax* peripheral parasitaemias.

Several approaches have been described for reducing human DNA contamination in malaria samples. Flow cytometry [16] and hybrid selection [17], [18] methods have demonstrated effective reduction of human DNA in stored blood and DNA samples, respectively, with promising prospects for analysis of archived samples. For higher sample throughput applications, in field settings where standard laboratory facilities are available for immediate sample processing, methods based on separation or filtration of the white blood cell (WBC) fraction from fresh whole blood offer a more practical and cost-effective approach [11], [15], [19]–[22]. The most cost-effective and perhaps most widely used filtration method utilises home-made cellulose fibre-based constructs (CF11 columns) [11], [20], [22]. Sriprawat and colleagues have described the efficacy of WBC depletion by CF11 filtration in *P. vivax* and demonstrated the method to be efficient at a cellular level [20]. However, the diploid human genome (∼6.4 Gb) within each WBC is >200 times larger than the haploid *P. vivax* genome (∼28 Mb) of an infected red blood cell (RBC), hence, the efficiency of this approach at the molecular level (percentage of human DNA and *P. vivax* DNA yield) was unclear. Dharia and colleagues applied CF11 filtration to produce a high *P. vivax* sequence yield on the Illumina Genome Analyzer (GA) platform [11], but despite a very high parasite density (∼100,000 parasites µl^−1^), approximately 50% of the sequence reads mapped to the human genome, raising uncertainty regarding the efficacy of their approach on lower parasitaemia infections.

Within the framework of genome-wide drug resistance studies, we addressed both human DNA contamination and *P. vivax* DNA yield at the molecular level in clinical isolates with a broad range of parasite densities. We present the efficacy of two sample preparation approaches, a) CF11 filtration alone (to deplete the human WBC content), and b) CF11 filtration followed by schizont maturation in short-term *ex vivo* culture (to enhance the parasite yield). Using the Illumina GAII platform, we assess the sequence yield and coverage distributions in 26 processed isolates including 24 pure *P. vivax* isolates and 2 mixed-species *P. vivax*+*P. falciparum* infections.

## Materials and Methods

### Sample Collection

Samples were collected from patients presenting with uncomplicated *P. vivax* malaria as determined by microscopy at the Royal Darwin Hospital, Australia (returning travellers), the Rumah Sakit Mitra Masayarat Hospital in Papua Indonesia, and the Shoklo Malaria Research Unit in Thailand. All samples were collected from patients presenting to outpatient clinics with *P. vivax* parasitaemia as determined by microscopy. Exclusion criteria included severe malaria, administration of anti-malarial or antibiotic treatment in the previous 2 weeks, and age less than 3 years. The volume of venous blood collected was tiered by patient age and weight. In children (age <5 years), blood volumes were specified as 1 ml blood per kg body weight, with a maximum of 6 mls. In children >5 years of age and adults, 12 and 18 mls were drawn, respectively. All blood samples were drawn into lithium heparin Vacutainer™ tubes (Becton-Dickinson).

### Ethical Approval

All biological samples and patient data were collected with written, informed consent from the patient or a parent or guardian. Ethical approval for this study was obtained from the Human Research Ethics Committee of the NT Department of Health & Families and Menzies School of Health Research, Darwin, Australia (HREC 09-83), the Eijkman Institute Research Ethics Commission (EIREC 2010-47), the Ethics Committee of the Faculty of Tropical Medicine, Mahidol University, Thailand (MUTM 2006-029) and the Oxford Tropical Research Ethics Committee, Oxford University, UK (OXTREC 27-05).

### White Blood Cell Depletion

All samples were processed within 6 hours of venepuncture. The plasma and buffy coat layer were removed after separation by centrifugation at 1,500–2,000 rpm for 10–15 minutes at ∼22°C. The remaining pellet was resuspended in an equal volume of phosphate-buffered saline (PBS) and subject to CF11 (Whatman) filtration, as previously described [20]. Briefly, the re-suspended blood was added to a CF11 column which had been pre-washed with PBS, and the RBCs were gently washed through with PBS until the liquid dripping into the collection tube was clear. The filtered blood sample was then centrifuged at 1000 rpm for 5 minutes, the supernatant was removed, and the pellet was re-suspended in an equal volume of PBS. The filtration process was then repeated with a new CF11 column. After the study was completed, Whatman discontinued the production of CF11. However, as discussed elsewhere [23], unpublished trials in *P. vivax* field isolates indicate that medium-sized cellulose fibre powders available from other companies (e.g. Sigma product number C6288), provide equivalent leukocyte retention to Whatman CF11. In a subset of the Indonesian and returning traveller samples, aliquots were collected at different stages of blood processing. A summary of the sample numbers available for each processing step and study site is presented in Table 1.

**Table 1:** Summary of Samples and Processing Methods.

Samples/Sites	Species	Preparation	Sequencing
4 Traveller (Darwin)	3 *P. vivax*	3 Unprocessed	–
		3 Post-CF11	^β^ 2
		α 3 Post-CF11+ Culture	3
	1 *P. vivax*+*P. falciparum*	1 Post-CF11	1
15 Indonesia	15 *P. vivax*	8 Post-CF11	–
		15 Post-CF11+ Culture	–
20 Thailand	19 *P. vivax*	19 Post-CF11+ Culture	19
	1 *P. vivax*+*P. falciparum*	1 Post-CF11	1
	**37 ** ***P. vivax***		**24 ** ***P. vivax*** ** (22 independent)**
	**2 ** ***P. vivax*** **+** ***P. falciparum***		**2 ** ***P. vivax*** **+** ***P. falciparum***

α One isolate failed culture. ^β^ Excluded sample aliquot from the isolate which failed culture.

### Schizont Maturation: Short-term *ex vivo* Culture

After double CF11 filtration, the parasites in the remaining RBC preparation were cultured *ex vivo* to enable early blood stages to mature to schizonts, as described elsewhere [24]. Incomplete culture medium was prepared with McCoy’s 5A medium supplemented with HEPES (25 mM), L-glutamine (2 mM), gentamicin (40 mg/L) and D-glucose (0.24%). Twenty percent AB+ human serum (inactivated at 56°C for 30 mins) was added to the incomplete medium to prepare the complete medium. The RBC pellet was added to complete medium at a 2% haematocrit. The resultant blood medium mixture was split accordingly into culture flasks (75 cm^3^) and incubated at 37.5°C for ∼40–48 hours, using a candle jar to control O_2_ and CO_2_ levels. After culture, the RBC pellet was obtained by centrifugation at 2,000 rpm for 10 minutes. Thick and thin blood films were made on pre-, peri- and post-culture samples in order to monitor the extent of parasite maturation in culture.

### DNA Extraction and Human and *P. vivax* DNA Quantification

DNA was extracted from the RBC pellets using QIAamp Blood Midi or Maxi kits (Qiagen) as per the manufacturer’s protocol. *Plasmodium* species was confirmed by PCR and real-time quantitative PCR (RT-qPCR) [25], [26]. Total sample DNA concentration was measured using Invitrogen’s Quant-iT™ dsDNA HS assay as per the manufacturer’s protocol.

The percentage of human (and *P. vivax*) DNA in the Indonesian and traveller samples was estimated by RT-qPCR with human (*Toll-like receptor 9* [*TLR9*]) and *P. vivax* (*P. vivax fructose 1, 6-bisphosphate aldolase* [*pvaldo*]*:* PVX_118251) specific primers. Primer sequences (5'-3') were as follows: *TLR9-*F: ACGTTGGATGCAAAGGGCTGGCTGTTGTAG, *TLR9-*R: ACGTTGGATGTCTACCACGAGCACTCATTC, *pvaldo-*F: GACAGTGCCACCATCCTTACC, *pvaldo-*R: CCTTCTCAACATTCTCCTTCTTTCC. The Indonesian samples were processed at the Eijkman Institute, and the traveller samples were processed at the Menzies School of Health Research. Cloned plasmid *TLR9* and *pvaldo* fragments surrounding the targeted RT-PCR amplicons were generated as standards for the assays using the TOPO TA Cloning® Kit (Invitrogen). The plasmids were diluted as appropriate to encompass the expected concentration range of the test samples. Separate SYBR® Green-based reactions comprising 1 µl DNA template, 2x SensiMix *No Ref* (Quantace), 200 nM each primer, and Nuclease-free water to a 10 µl final volume, were prepared for each of the *TLR9* and *pvaldo* assays and run on a Corbett Rotor-Gene 6000 machine with the following cycle conditions: 1×95°C/15 mins; 40 × (95°C/30 sec; 60°C/30 sec; 72°C/45 sec); 1×68°C - 90°C in 0.5°C increments. All samples and standards were run in duplicate. The concentration of the test sample’s *TLR9* and *pvaldo* amplicons were approximated by standard curve analysis using the Corbett Rotor-Gene 6000 series software 1.7. In order to calculate the relative proportions of human and *P. vivax* DNA, the estimated concentration of *P. vivax* DNA was multiplied by a factor of ∼0.01 to account for the relative differences in the size of the *TLR9* (4550 bp) and *pvaldo* (3115 bp) plasmids, and the human (∼3.2 Gb) and *P. vivax* (∼28 Mb) genomes. The percentage of human DNA was calculated using the adjusted *P. vivax* DNA quantity.

In the Thai samples, the percentage of human DNA was estimated at the Wellcome Trust Sanger Institute using a RT-qPCR assay with human standards and primers [19]. The *P. vivax* DNA quantity was then extrapolated as the total DNA quantity minus the estimated human DNA quantity.

### Statistical Analysis

Excluding the mixed-species infections, the significance of the difference between Indonesia (*n* = 15) and Thailand (*n* = 19) in each of *ex vivo* culture duration, percentage of schizonts post-CF11+ culture, percentage of human DNA post-CF11+ culture, and *P. vivax* DNA yield post-CF11+ culture was assessed using the Mann-Whitney U test.

Using the Indonesian isolates for which both pre- and post-culture aliquots were available (*n* = 8), the Wilcoxon signed-rank test was used to assess the difference between paired pre- and post-culture isolates in each of human DNA percentage and *P. vivax* yield.

Comparisons between the RT-qPCR and Illumina estimates of human DNA percentage were undertaken on data derived from the post-CF11+ culture aliquots of the pure *P. vivax* isolates (*n* = 22 [3 Traveller, 19 Thailand]) for which Illumina sequence was available (see Table 1). The correlation between the RT-qPCR and Illumina human DNA percentage estimates was assessed using Spearman’s rank correlation coefficient. The significance of the difference in magnitude between the percentage of human DNA determined by RT-qPCR and Illumina was assessed using the Wilcoxon signed-rank test. Significance for all tests was determined at the 5% threshold. All analyses were undertaken using R software [27].

### Whole Genome Sequencing: Library Preparation and Sequencing

Twenty-four pure *P. vivax* and 2 *P. vivax*+*P. falciparum* infections (see Table 1) were sequenced on the Illumina GA II or HiSeq 2000 platform (see Table S1) (http://www.illumina.com/systems/genome_Analyzer.ilmn) at the Wellcome Trust Sanger Institute. Pre- and post-culture aliquots were sequenced for two of the pure *P. vivax* isolates, leaving 22 independent samples (see Table 1). Paired-end multiplex or non-multiplex (see Table S1) libraries were prepared from 500 ng - 1 µg total DNA, as per the manufacturer’s protocol, with the exception that genomic DNA was fragmented using Covaris Adaptive Focused Acoustics rather than nebulisation. Multiplexes comprised 12 tagged samples. Cluster generation and sequencing were undertaken as per the manufacturer’s protocol for paired-end 75 bp, 76 bp or 100 bp sequence reads (see Table S1).

### Sequence Alignment and Coverage Statistics

The total yield of raw, unaligned, sequence data was recorded for each sample. The data was then aligned against each of the *P. vivax* Sal-1 (PlasmoDB release 9.1 [http://plasmodb.org/common/downloads/release-9.1/PvivaxSaI1/fasta/data/PlasmoDB-9.1_PvivaxSaI1_Genome.fasta] [10], and NCBI accession AB649419.1 [apicoplast genome: http://www.ncbi.nlm.nih.gov/nuccore/AB649419.1] [28]), *P. falciparum* (3D7 PlasmoDB release 9.1 (apicoplast genome included) [http://plasmodb.org/common/downloads/release-9.1/Pfalciparum3D7/fasta/data/PlasmoDB-9.1_Pfalciparum3D7_Genome.fasta] [29]), and *Homo sapiens* (NCBI build V37 [30]) reference genomes using the *bwa* read mapping algorithm (version 0.6.2), with the default execution parameters (available from http://bio-bwa.sourceforge.net) [31]. The percentage of reads mapping against each of the *P. vivax*, *P. falciparum*, and human genomes was derived from the resultant *bam* alignment files and recorded for each sample [32]. The *P. vivax bam* files (and *P. falciparum bam* files for the 2 mixed-species samples) were analysed further with the ‘depth’ command provided in the SAMtools application (version 0.1.18) to compute the per-base depth. The default SAMtools depth parameters were used with the exception of the mapping quality threshold (Q) of 20, corresponding to 1% mapping error). The resultant depth files were processed using R software [27] to derive summary statistics on sequence depth (fold-coverage of a given region) and breadth (percentage of a given region covered by a given number of reads). Breadth statistics were derived for a minimum of 1 and of 20 reads. Breadth and depth statistics were determined for the full nuclear reference genome (i.e. excluding the mitochondrial and apicoplast sequences) and coding and non-coding regions. Coding and non-coding positions were defined in each of *P. vivax* and *P. falciparum* with the Sal-1 PlasmoDB release 9.1 (http://plasmodb.org/common/downloads/release-9.1/PvivaxSaI1/gff/data/PlasmoDB-9.1_PvivaxSaI1.gff) and 3D7 PlasmoDB release 9.1 [http://plasmodb.org/common/downloads/release-9.1/Pfalciparum3D7/fasta/data/PlasmoDB-9.1_Pfalciparum3D7_Genome.fasta] annotation files, respectively. Summary plots were generated using R software [27].

## Results

### Samples

**Figure 1:**
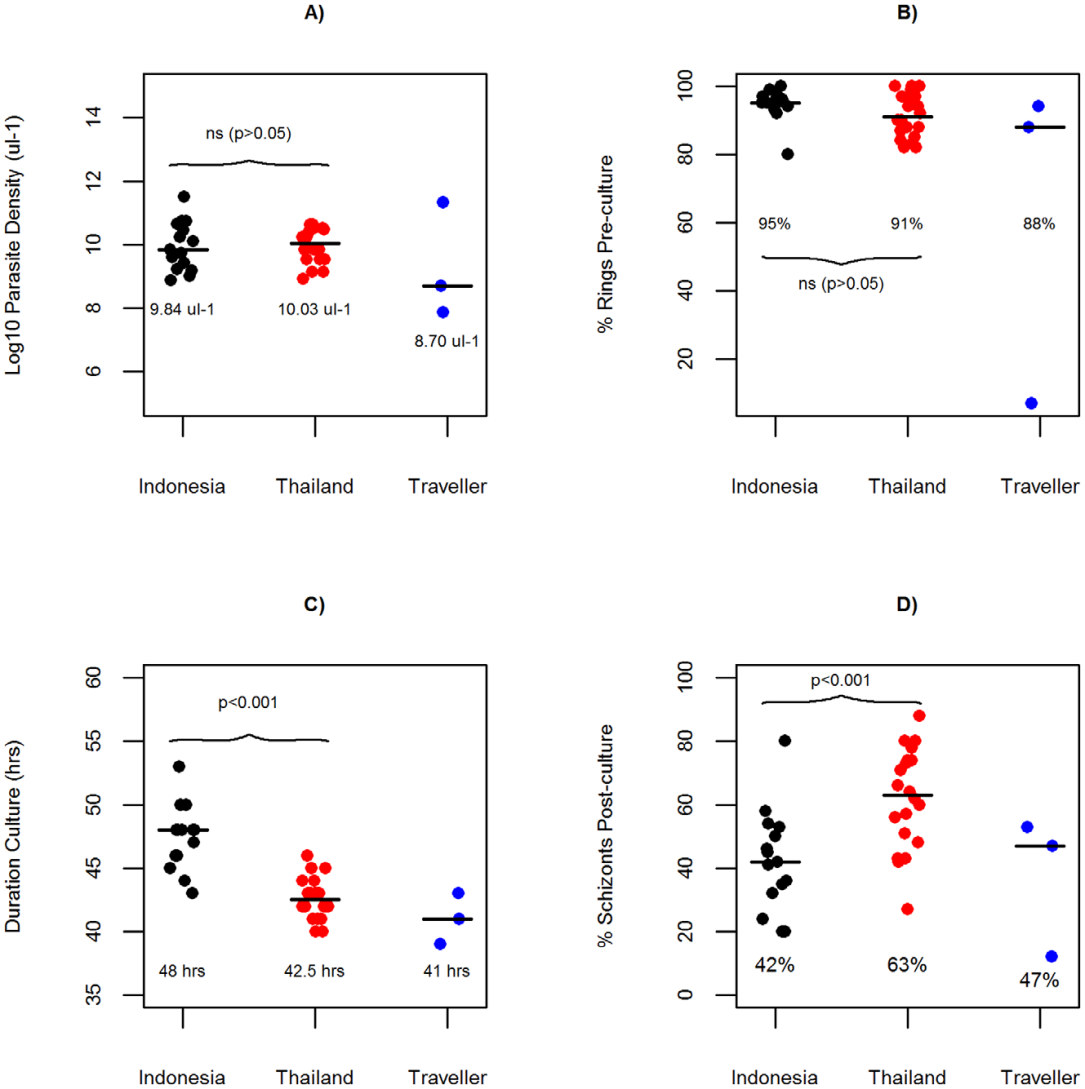
Distribution by sample origin of A) parasite density, B) % ring stage parasites pre-culture, C) duration of *ex vivo* schizont maturation, and D) % schizont stage parasites post-culture. Vertical lines indicate median of distribution.

In total, 39 independent *P. vivax* isolates were processed: 4 from returning travellers, 15 from Indonesian patients and 20 from Thai patients (Table 1). One of the returning traveller isolates (Papua New Guinea) and one of the Thai isolates were found to be mixed-species infections of *P. vivax* and *P. falciparum* by PCR and thus were not included in the sample preparation analyses. In the remaining 37 pure *P. vivax* samples, parasite densities ranged from 2,600 to 99,480 parasites µl^−1^, with a median of 18,640 parasites µl^−1^ (Figure 1A). Sampling was undertaken largely within the framework of a complementary study investigating *P. vivax ex vivo* drug susceptibility testing, which requires processing predominantly ring stage parasites (see [24]). Therefore the majority of samples in the current molecular study exhibited a high proportion of ring stage parasites (median 94% [range: 7–100%]) (Figure 1B). Short-term *ex vivo* culture was successful in all except one sample (DRW-002), the median duration of incubation being 43.5 hours [range: 39–53] with a final median percentage of schizonts of 52% [range: 12–88%] (Figure 1C and D). The median incubation period in the Thai samples (42.5 hours [range: 40–46]) was significantly shorter than in the Indonesian samples (median = 48 hours [range: 43–53) (Mann-Whitney U test: *p* = 5.6×10^−7^). In addition, the Thai samples produced a greater percentage of schizont stages (median = 63% schizonts [range: 27–88]) than the Indonesian samples (median = 42% [range: 20–80]) (Mann-Whitney U test: *p* = 6×10^−7^). Details on the clinical and laboratory properties of each sample are presented in (Table S2).

### Percentage Human DNA (RT-qPCR)

**Figure 2:**
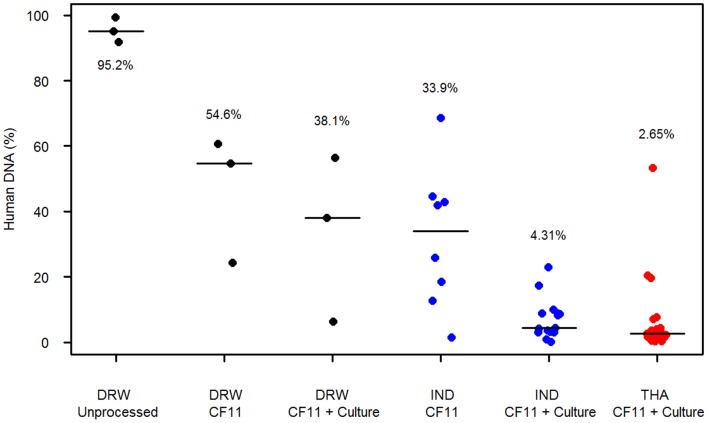
Distribution of human DNA quantity (%) by sample origin and blood processing step. Vertical lines indicate median of distribution.

In each of the Indonesian, Thai and traveller sample sets, the distribution of human DNA percentages (as defined by RT-qPCR) at each processing step is illustrated in Figure 2. The human DNA contamination assessed in three samples from travellers prior to processing showed all samples to have >90% human DNA. After CF11 filtration, human DNA contamination fell from a median of 95.2% [range: 91.9–99.4] to 54.6% [range: 24.3–60.6]. This dropped further to a median of 38.1% [range: 6.29–56.5] following short-term culture.

The impact of the short term culture step relative to CF11 filtration alone was further assessed in 8 Indonesian isolates with paired pre- and post-culture aliquots, and found to reduce human DNA by 4.91-fold [range: 1.87–13.48]; from a median of 33.9% [range: 1.58–68.6] in post-CF11 samples to 6.22% [range: 0.20–23.0] following culture (*p* = 0.008). Of the 3 traveller and 8 Indonesian samples, 81.8% (9/11) of the post-culture aliquots exhibited less than 30% human DNA, in contrast to 45.5% (5/11) of the post-CF11 aliquots.

In the full set of 37 post-CF11+ culture samples, the median human DNA content was 3.85% [range: 0.20–56.5]. The median human DNA percentage in the post-CF11+ culture Thai samples was 2.65% [range: 0.40–53.4]. This was lower than in the full set of 15 post-CF11+ culture Indonesian samples (4.31% [range: 0.20–44.6]) but the difference was not significant (*p* = 0.086).

### *P. vivax* DNA Yield

**Figure 3:**
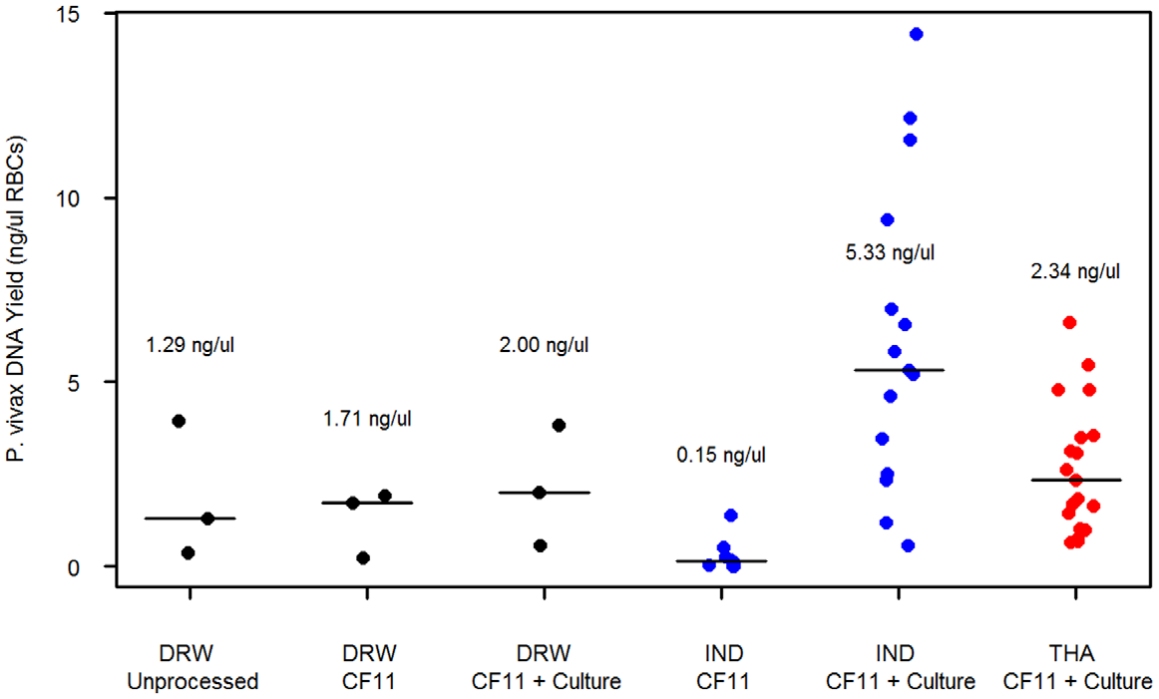
Distribution of *P. vivax* yields (ng µl^−1^ packed RBCs) by sample origin and blood processing step. Vertical lines indicate median of distribution.

In each of the Indonesian, Thai and traveller sample sets, the distribution of the *P. vivax* DNA yield at each processing step is illustrated in Figure 3. In the 3 unprocessed samples from returning travellers, the median *P. vivax* DNA yield was 1.29 ng µl^−1^ packed red blood cells (pRBCs)[range: 0.36–3.93], rising to 1.71 ng µl^−1^ pRBCs [range: 0.24–1.91] following CF11 filtration, and 2.00 ng µl^−1^ pRBCs [range: 0.57–3.82] following the addition of short term culture.

In the 8 Indonesian isolates with paired pre- and post-culture aliquots, the *P. vivax* DNA yield increased a median 34-fold from a median of 0.15 ng µl^−1^ pRBCs [range: 0.01–1.39] in the post-CF11 aliquots to a median of 6.2 ng µl^−1^ pRBCs [range: 2.34–14.4] after additional culture (*p* = 0.001). Extrapolating from the yield estimates per microliter pRBCs, in a “standard” 5 ml (50% haematocrit) whole blood sample, amongst the 3 traveller and 8 Indonesian isolates, 100% of the post-culture aliquots would give a sufficient *P. vivax* DNA yield (≥500 ng) for Illumina genome sequencing compared to 54.5% (6/11) of the post-CF11 aliquots. Including the human DNA yield, 72.7% (8/11) of the post-CF11 aliquots would yield sufficient total DNA for whole genome sequencing. Considering both the yield of total DNA (500 ng threshold) and percentage of human DNA (30% threshold), 81.8% (9/11) of the post-culture aliquots would meet the requirements for whole genome sequencing in contrast to 36.4% (4/11) of the post-CF11 aliquots.

In the full set of 37 post-CF11+ culture samples, the median *P. vivax* DNA yield was 3.12 ng µl^−1^ pRBCs [range: 0.56–14.4]. The median yield in the Indonesian samples (5.33 ng µl^−1^ pRBCs [range: 0.56–14.4]) was significantly higher than in the Thai samples (2.34 ng µl^−1^ pRBCs [range: 0.64–6.61]) (*p* = 0.010).

### Sequence Data: Yield and Alignment

**Figure 4:**
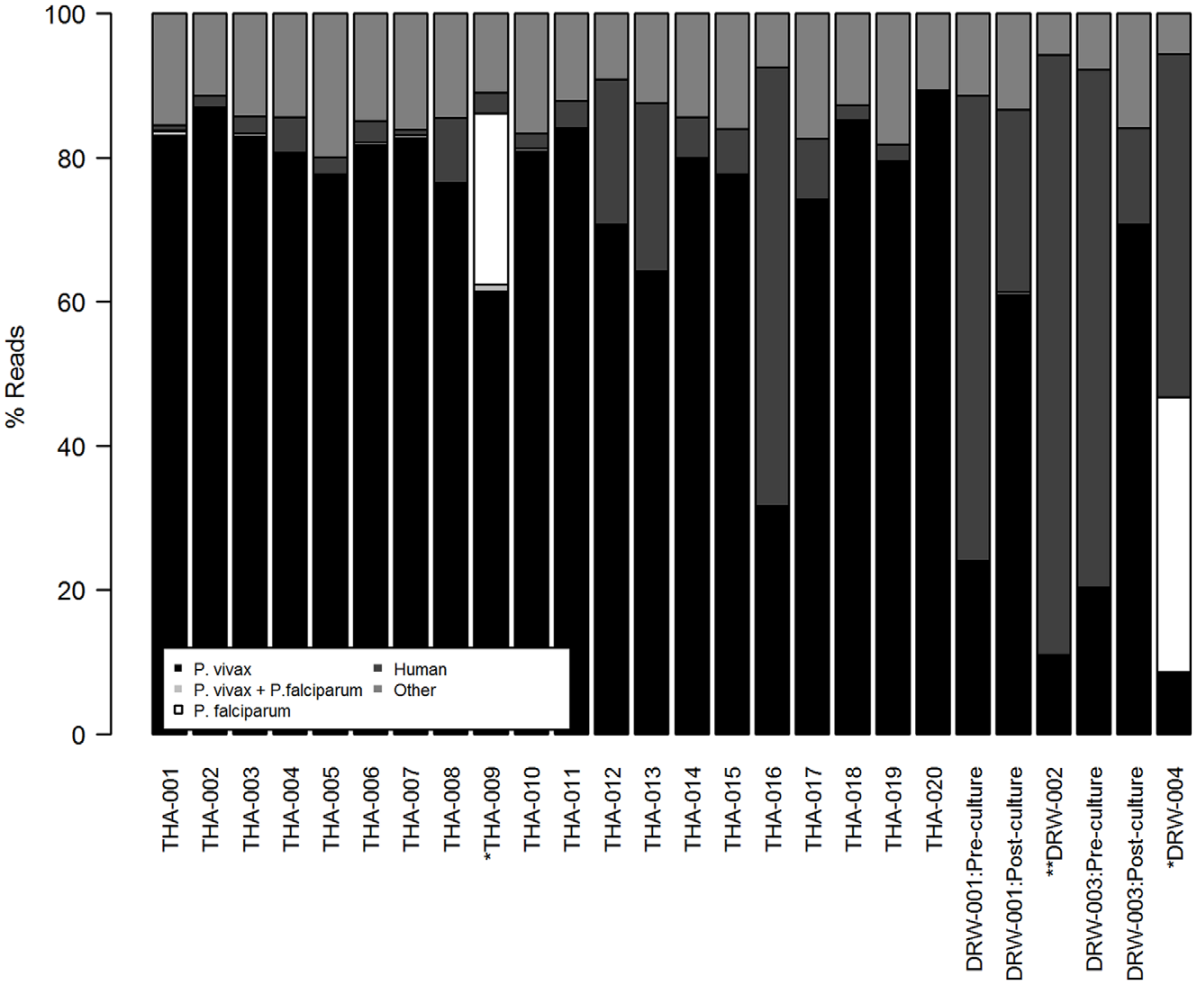
Distributions of Illumina Read Alignments against the *P. vivax*, Human, and *P. falciparum* Reference Genomes. *Mixed-species confirmed by PCR. **Poor maturation in *ex vivo* culture. Note: % *P. vivax* reads does not include reads which cross-map against *P. falciparum.*

**Figure 5:**
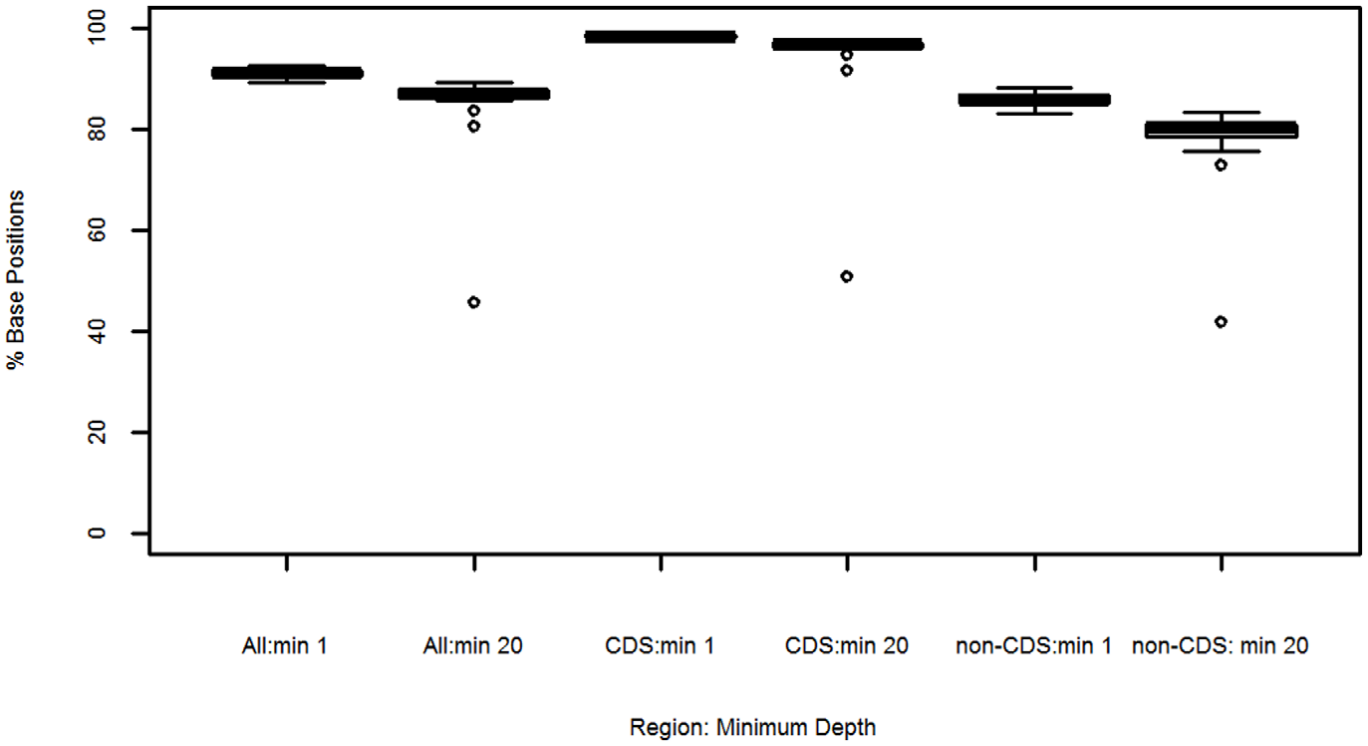
Sequence Depth Distributions across the *P. vivax* Sal-1 Reference Genome and in Coding and Non-coding Regions. Samples = 22 independent, pure *P. vivax* isolates. CDS = coding sequence.

**Figure 6:**
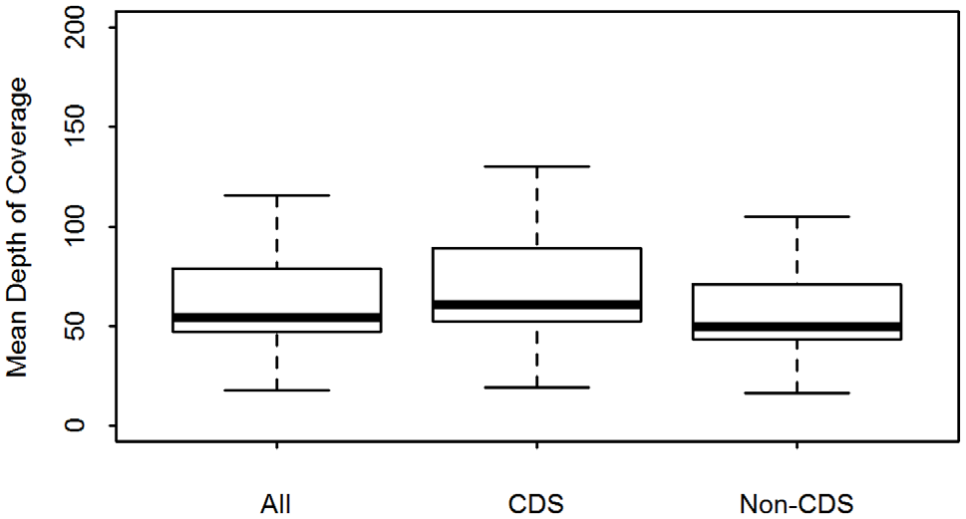
Sequence Breadth Distributions across the *P. vivax* Sal-1 Reference Genome and in Coding and Non-coding Regions. Samples = 22 independent, pure *P. vivax* isolates. CDS = coding sequence.

Sequence data was successfully generated on 22 post-CF11+ culture *P. vivax* isolates, 2 post-CF11 (pre-culture) *P. vivax* sample aliquots, and 2 mixed-species *P. vivax*+*P. falciparum* isolates. The data are available in the European Nucleotide Archive (ENA) (http://www.ebi.ac.uk/ena/data/search?query=plasmodium) [33]. For ethical purposes, the data submitted to the ENA are filtered to remove the human alignments. Sample accession numbers for the raw data are presented in (Table S3). Details of the sequence yield and mapping statistics for each sample are presented in (Table S1). The median yield of total sequence data generated was 2321 million bases (Mb) [range: 902–6911 Mb]. The observed variation in total sequence yield may reflect variation in a range of factors including read length, platform (Illumina GAII versus HiSeq) and multiplex versus non-multiplex sequencing (see Table S1). Figure 4 presents bar-plots illustrating the distribution of reads which aligned against each of the *P. vivax*, human, and *P. falciparum* reference genomes, as well as “other”, non-aligned reads, in each sample. In the 22 independent, pure *P. vivax* samples, the median percentage of reads which mapped to the *P. vivax* reference was 79.7% [range: 11.0–89.1]. Across these samples, the median sequence depth was 54X [range: 18–116X], as illustrated in Figure 5. In terms of read breadth, the median percentage of the *P. vivax* reference covered by a minimum of 1 base was 90.9% [range: 89.1–92.6%], dropping to a median of 86.9% [range: 45.6–89.1%] covered by a minimum of 20 bases (Figure 6).

As expected, the percentage of reads which mapped against the human reference was positively correlated with the percentage of human DNA estimated by RT-qPCR (rho = 0.94, *p* = 4.7×10^−11^) in the 22 independent pure *P. vivax* isolates. The percentage of human reads was only a median 1.34-fold [range: 0.35–4.02-fold] higher than the RT-qPCR estimates of human DNA. However, the difference between the median percentage of human reads (4.40% [range 0.18–83.2%]) and RT-qPCR human DNA estimates (2.7% [range 0.40–56.5%]) was significant (*p* = 0.0053). Also as expected, the post-CF11 aliquots of DRW-001 and DRW-003 demonstrated a substantially higher median percentage of human reads (2.55 and 5.36-fold difference, respectively) and accordingly lower median *P. vivax* read depth (2.14 and 3.76-fold difference, respectively) than their respective post-culture aliquots (Table 1).

In the 22 independent, pure *P. vivax* samples, a median of 0.30% reads [range: 0.17–0.61%] mapped to both the *P. vivax* Sal-1 and *P. falciparum* 3D7 reference genomes. The median percentage of reads which did not map to the human, Sal-1 or 3D7 reference genomes (“other”) in these samples was 14.4% [range: 5.79–20.0%].

### Sequence Data: Uniformity of Coverage

**Figure 7:**
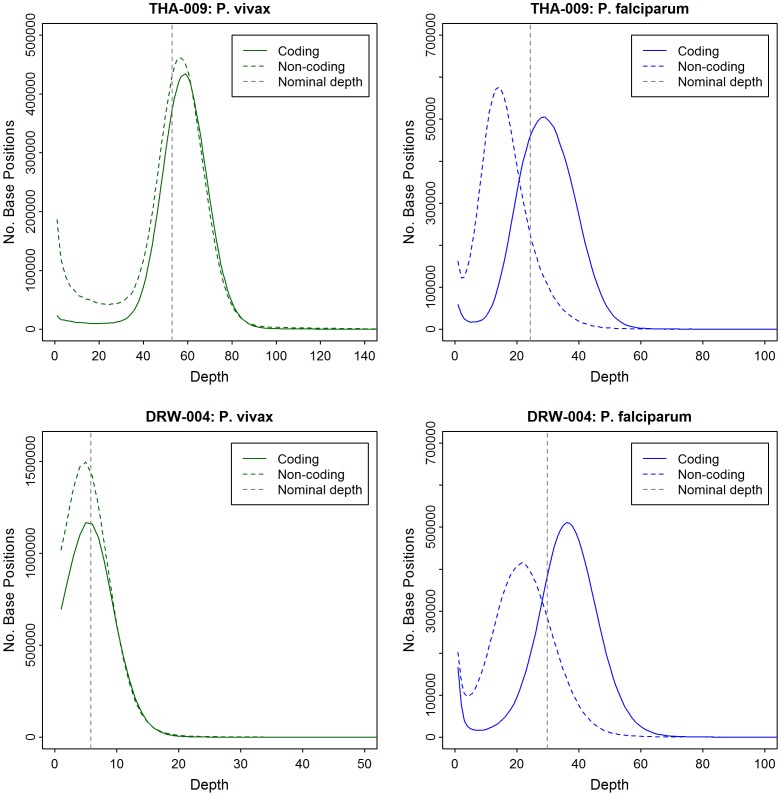
Comparative Sequence Depth Distributions between the *P. vivax* and *P. falciparum* Genome in THA-009 and DRW-004. Plots indicate the number of bases (y-axis) with a given sequence depth (x-axis) in different regions of the *P. vivax* (green) and *P. falciparum* (blue) genome. Whilst the *P. vivax* coding and non-coding sequences peak close to the nominal total sequence depth, the *P. falciparum* coding and non-coding sequences peak at moderately different coverage depths.

The coding and non-coding sequence depth distributions for *P. falciparum* and *P. vivax* in the 2 mixed-species sample are presented in Figure 7. In both samples, the *P. vivax* non-coding and coding sequences peaked at similar depths of coverage (56 and 59 respectively in THA-009; both 5 in DRW-004), close to the expected (nominal) sequence depths (53.0 and 5.5 in THA-009 and DRW-004, respectively). In contrast, the *P. falciparum* non-coding sequences peaked at moderately lower depth than the corresponding coding sequences (14 and 29 respectively in THA-009; 22 and 36 respectively in DRW-004) and nominal sequence depths (24.3 and 29.8 in THA-009 and DRW-004, respectively). The coding and non-coding coverage distributions in the 22 pure *P. vivax* samples (Figure S1) confirmed this reduced coverage bias across the *P. vivax* genome.

## Discussion

Limited parasite DNA and extensive human DNA contamination present major challenges to high-throughput genomic analysis of clinical *P. vivax* isolates. We demonstrate the efficacy of a simple two-step CF11 filtration+schizont maturation method in yielding samples with low human contamination and high parasite yield. Using the Illumina GAII platform, we confirm high *P. vivax* sequence yield and uniform coverage across the genome in natural patient isolates prepared using this two-step method. Additional sequence data from 2 mixed *P. vivax*+*P. falciparum* infections demonstrated potential for accurate WGS analysis of both species in mixed infections.

The simplicity and low resource intensity of the CF11 filtration procedure was highly practical for processing isolates in field settings. However, in 8 paired Indonesian isolates, the filtration step alone provided only moderate reduction in human DNA percentage (to a median of 33. 9%) and resulted in a low *P. vivax* DNA yield (median 0.15 ng µl^−1^ pRBCs). The additional process of short-term *ex vivo* culture, whilst less simple and requiring more resources than filtration alone, reduced the percentage of human DNA to a median of 6.22% and increased the parasite DNA yield to a median of 6.2 ng µl^−1^ pRBCs. It should be noted that the short-term culture step facilitates sample preparation not only through enhancement of *P. vivax* yield but also through further depletion of the remaining WBCs which have been observed to adhere to the internal surfaces of plastic culture flasks (unpublished data).

A minimum of 500 ng DNA generally ensures effective library preparation on the Illumina platform. Libraries have been prepared with lower quantities but the risk of failure increases with decreasing DNA quantity. In a study addressing Illumina sequence coverage in WBC-depleted *P. falciparum* field isolates, high sequence yields (>40X) were observed in samples with ≤30% human DNA [19]. In our study, a third of the post-CF11 samples exhibited ≤30% human DNA and ≥500 ng total DNA, in contrast to more than 80% of the corresponding post-CF11+ culture samples. Therefore, the two-step CF11+ schizont maturation approach ensures a greater probability of successful *P. vivax* sample preparation for high-throughput genome sequencing. However, with continual improvements in sequencing technologies, the effective human DNA percentage and parasite yield thresholds are anticipated to rise and fall, respectively, improving the prospects for adopting processing methods that apply CF11 filtration alone. In field settings where even CF11 filtration is not feasible, developments in DNA-based parasite enrichment methods such as hybrid selection may present an alternative approach [17], [18].

A potential limitation of the two-step approach is failure of a sample to mature in *ex vivo* culture. In our study, possibly as a result of patient blood sampling at the peak of a paroxysm (41.9°C), one isolate in a returning traveller failed to mature in short-term culture. As a result, the difference in the percentage of human DNA between the post-CF11 (60.63%) and post-CF11+ culture (56.48%) samples was negligible. Furthermore, in contrast to the other 10 sample pairs with successful maturation, *P. vivax* DNA yield (ng µl^−1^ packed RBCs) was greater post-CF11 (1.71) than post-CF11+ culture (0.57). This apparent loss of parasite DNA is likely to reflect parasite death and DNA degradation during the culture step. Therefore, early termination of culture is advised following signs of extensive parasite death.

As demonstrated in comparisons between the Thai and Indonesian samples, variation in maturation efficacy may be observed between isolates from different origins. Whether this reflects underlying differences in the biology of *P. vivax* isolates from different geographic origins remains to be determined. The efficacy of short-term *P. vivax* culture thus requires further assessment in a broader geographic range of isolates.

Using the Illumina GAII and HiSeq platforms on 22 pure *P. vivax* samples prepared using the two-step method, we observed high depth (median 54-fold coverage) and breadth (median 90.9% and 86.9% at minimum depth 1 and 20, respectively) of coverage across the Sal-1 reference genome. Although variation was observed in the percentage of reads which aligned against the Sal-1 reference (11–89.1%), the median was moderately high at 79.7%. A large proportion of this variation presumably reflected the percentage of human reads, which ranged from 0.18 to 83.2%, emphasizing the importance of effective sample preparation. The cause of the moderate inflation (median 1.34-fold increase) in the percentage of human reads relative to the percentage of starting human DNA remained unclear. A small degree of error in the human quantification method, or bias in favour of human sequence during the library preparation, cluster generation and/or sequencing process might also explain the inflation. The latter should add further demand for effective sample preparation.

In the 22 pure *P. vivax* isolates, a low proportion of reads (0.17–0.61%) aligned against both the *P. vivax* and *P. falciparum* reference genomes. The low frequency of these reads is promising for whole genome analyses of mixed *P. vivax*+*P. falciparum* infections. However, further characterisation in these regions is required, including assessment of sequence complexity and gene ontology, and addressing the influence of varying read mapping thresholds. The ability to analyse mixed-species infections is critical in enabling assessment of the reservoir of *P. vivax* isolates within mixed-species infections in regions with co-endemic *P. vivax* and *P. falciparum*. Increasing Illumina read lengths should facilitate further resolution of the species in mixed infections. In the mixed species samples investigated here, the proportion of *P. vivax* relative to *P. falciparum* isolates were moderate and thus reasonable coverage of the *P. vivax* genome was observed. However, in many mixed-species infections, *P. vivax* appears to be suppressed by *P. falciparum* and hence, the density of the former may be very low [34], [35]. In such samples, short-term culture under conditions favouring *P. vivax* maturation may be advisable. Further studies are required to identify optimal methods for preparing mixed-species infections for WGS.

In the 22 pure *P. vivax* isolates, a consistent proportion of reads, ranging from 5.79–20.0%, did not align against the *P. vivax*, human or *P. falciparum* reference. Further investigations are required to identify the nature of these “other” reads. A proportion of the reads may represent highly diverse regions of the genome. Genomic rearrangements and deletions relative to the Sal-1 reference, and sequences overlapping currently unaligned contigs in the Sal-1 reference genome may also account for a proportion of these reads.

In *P. falciparum*, non-coding regions of the genome generally exhibit moderately lower read depth than coding-regions on the Illumina platform [15], [36]. With genome-wide sequence data from 2 mixed-species *P. vivax*+*P. falciparum* infections, we had the opportunity to compare the coverage distribution in coding versus non-coding regions between the two species with data generated under the same library preparation, cluster generation and sequencing conditions. In contrast to *P. falciparum*, presumably owing to the lower A+T content in the *P. vivax* genome (∼58% versus ∼81% in *P. falciparum*), the read depth frequency distributions in coding and non-coding regions both peaked close to the expected (nominal) sequence depth. The notable uniformity of coverage across the *P. vivax* genome will facilitate the detection of copy number variants, which have been shown to underlie important disease-causing or drug resistance mechanisms [37]–[39]. Furthermore, the ability to access non-coding regions of the genome using high-throughput sequencing methods is promising for SNP identification in these regions, which may carry important determinants of gene expression. In depth SNP analysis is beyond the scope of the current study, and will be addressed in a separate, larger scale multi-centre study of *P. vivax* diversity.

In conclusion, our study describes the efficacy of two alternative methods for preparing *P. vivax* field samples for high-throughput WGS, using currently available technology. The one-step method offers a more simple and practical approach, but exhibited only moderate efficacy in reducing human DNA, and limited parasite DNA yield. The two-step method was more efficient at reducing human DNA contamination and increasing parasite DNA yield, although at a greater resource cost. A video demonstration of these methods is now available online [40]. The demonstration of high depth, breadth and uniformity of coverage across both coding and non-coding regions of the genome in effectively prepared *P. vivax* field isolates provides an essential opportunity for unbiased exploration of the natural diversity in *P. vivax* populations.

## Supporting Information

Figure S1 **Coding and Non-coding Sequence Depth Distributions in 22 Independent, Pure** ***P. vivax*** **Isolates.** Plots indicate the number of bases (y-axis) with a given sequence depth (x-axis) in coding (solid green line) and non-coding (dashed green line) regions of the *P. vivax* genome. Dashed grey line indicates the expected (nominal) sequence depth for each sample.
(TIFF)

Table S1 **Sequence Coverage Statistics.** Pv = *P. vivax*; Pf = *P. falciparum*; ^a^ Multiplexes comprise 12 samples. ^b^ Quantitative real-time PCR estimate; ^c^ Reads overlapping *P. vivax* and *P. falciparum*.^ d^ Expected sequence depth at each position in the *P. vivax* genome – calculated as total number of bases sequenced divided by number of bases in the genome (based on Sal-1 reference genome size ∼27 Mb). ^e^ Percentage Sal-1 reference genome covered at given read depth. *Poor maturation during short-term culture.
(DOCX)

Table S2 **Clinical and Laboratory Sample Properties.** *Poor maturation during short-term culture.
(DOCX)

Table S3 **European Nucleotide Archive Sample Accession Numbers.** For ethics purposes, all samples were subject to human read alignment filtration prior to submission to the ENA.
(DOCX)
