# Supplementary figures and images for: Effective Preparation of Plasmodium vivax Field Isolates for High-Throughput Whole Genome Sequencing
Source: PLoS One. 2013 Jan 4;8(1):e53160. doi: 10.1371/journal.pone.0053160 (PMC3537768; doi:10.1371/journal.pone.0053160)

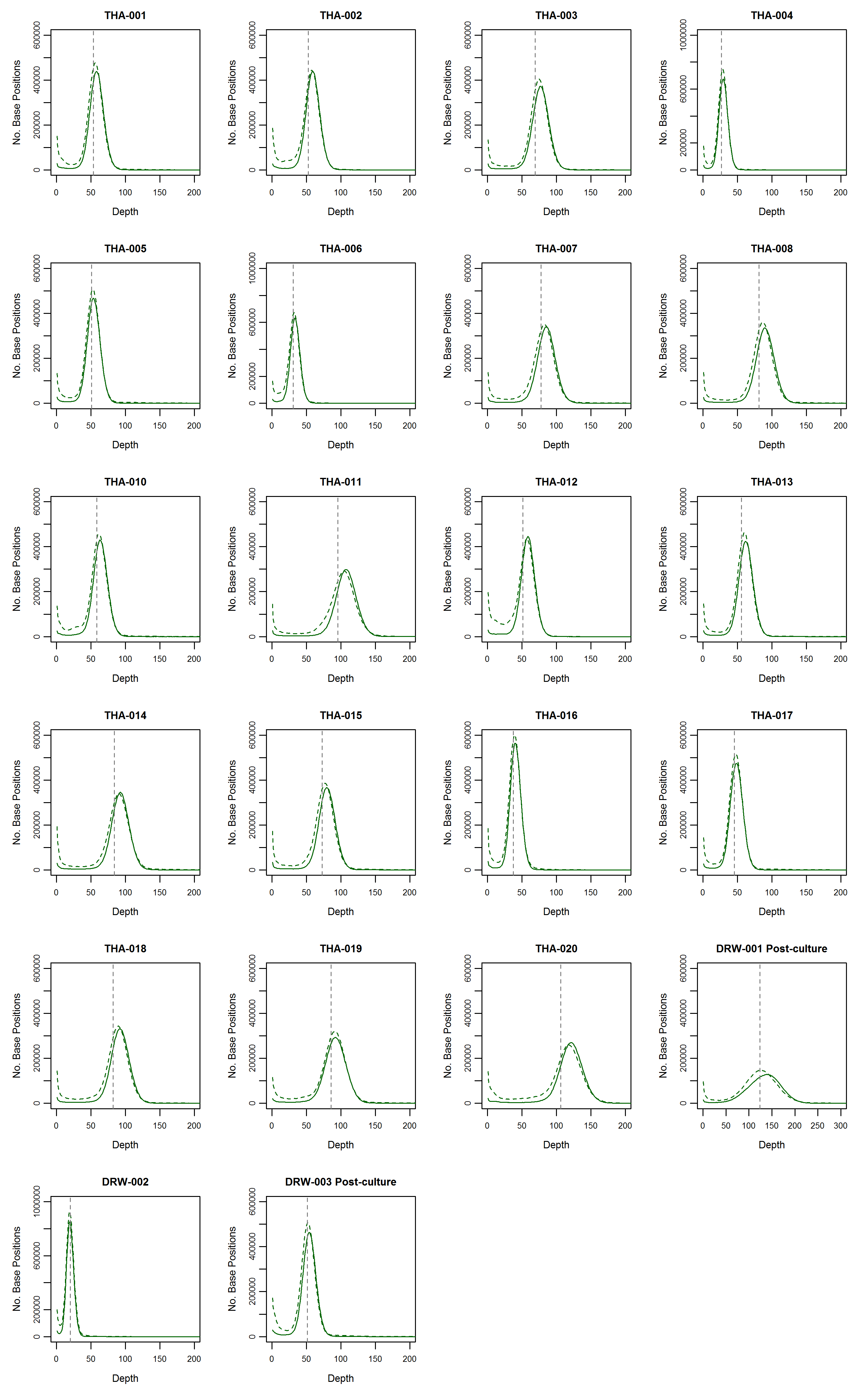

Supplement: Figure S1 — Coding and Non-coding Sequence Depth Distributions in 22 Independent, Pure P. vivax Isolates. Plots indicate the number of bases (y-axis) with a given sequence depth (x-axis) in coding (solid green line) and non-coding (dashed green line) regions of the P. vivax genome. Dashed grey line indicates the expected (nominal) sequence depth for each sample. (TIFF) [file pone.0053160.s001.tiff]
