# Supplementary material for: Effective Preparation of Plasmodium vivax Field Isolates for High-Throughput Whole Genome Sequencing
Source: PLoS One. 2013 Jan 4;8(1):e53160. doi: 10.1371/journal.pone.0053160 (PMC3537768; doi:10.1371/journal.pone.0053160)
Supplement: Table S1 — Sequence Coverage Statistics. Pv = P. vivax; Pf = P. falciparum; a Multiplexes comprise 12 samples. b Quantitative real-time PCR estimate; c Reads overlapping P. vivax and P. falciparum. d Expected sequence depth at each position in the P. vivax genome – calculated as total number of bases sequenced divided by number of bases in the genome (based on Sal-1 reference genome size ∼27 Mb). e Percentage Sal-1 reference genome covered at given read depth. *Poor maturation during short-term culture. (DOCX) [file pone.0053160.s002.docx]

**Table S1: Sequence Coverage Statistics**

| Sample | Species **^a^** | Processing | Platform | Read length (bp) | Multiplex **^b^** | Total Sequence Yield (Mb) | % Pv reads | % Pv +Pf reads **^c^** | % Pf reads | % Human reads | % "Other" reads | Pv read depth **^d^** | Pv read breadth: 1**^e^** | Pv read breadth: 20 **^e^** |
| --- | --- | --- | --- | --- | --- | --- | --- | --- | --- | --- | --- | --- | --- | --- |
| THA-001 | Pv | CF11 + Culture | HiSeq | 100 | Yes | 1729 | 83.7 | 0.61 | - | 0.81 | 15.5 | 50.6 | 90.9 | 86.5 |
| THA-002 | Pv | CF11 + Culture | HiSeq | 100 | Yes | 1636 | 87.0 | 0.27 | - | 1.59 | 11.4 | 50.5 | 91.2 | 86.1 |
| THA-003 | Pv | CF11 + Culture | HiSeq | 100 | Yes | 2248 | 83.3 | 0.32 | - | 2.36 | 14.3 | 66.0 | 89.7 | 86.5 |
| THA-004 | Pv | CF11 + Culture | HiSeq | 100 | Yes | 902 | 80.7 | 0.37 | - | 4.95 | 14.3 | 25.8 | 89.1 | 80.6 |
| THA-005 | Pv | CF11 + Culture | HiSeq | 100 | Yes | 1768 | 77.7 | 0.29 | - | 2.32 | 20.0 | 48.1 | 90.8 | 87.1 |
| THA-006 | Pv | CF11 + Culture | HiSeq | 100 | Yes | 1018 | 82.1 | 0.38 | - | 3.00 | 14.9 | 29.3 | 91.6 | 83.6 |
| THA-007 | Pv | CF11 + Culture | HiSeq | 100 | Yes | 2527 | 83.2 | 0.38 | - | 0.67 | 16.2 | 73.8 | 91.1 | 87.7 |
| THA-008 | Pv | CF11 + Culture | HiSeq | 100 | Yes | 2882 | 76.5 | 0.27 | - | 9.00 | 14.5 | 77.7 | 90.6 | 87.4 |
| THA-009 | Pv + Pf | CF11 + Culture | HiSeq | 100 | Yes | 2299 | 62.3 | 0.90 | 23.8 | 2.90 | 11.0 | 50.4 | 93.1 | 86.8 |
| THA-010 | Pv | CF11 + Culture | HiSeq | 100 | Yes | 1944 | 81.3 | 0.34 | - | 2.02 | 16.7 | 55.5 | 92.1 | 88.0 |
| THA-011 | Pv | CF11 + Culture | HiSeq | 100 | Yes | 3071 | 84.0 | 0.36 | - | 3.85 | 12.1 | 91.1 | 90.5 | 87.4 |
| THA-012 | Pv | CF11 + Culture | HiSeq | 100 | Yes | 1959 | 70.7 | 0.25 | - | 20.1 | 9.17 | 49.3 | 92.6 | 85.6 |
| THA-013 | Pv | CF11 + Culture | HiSeq | 100 | Yes | 2342 | 64.3 | 0.24 | - | 23.3 | 12.4 | 53.2 | 89.5 | 86.0 |
| THA-014 | Pv | CF11 + Culture | HiSeq | 100 | Yes | 2845 | 80.0 | 0.30 | - | 5.63 | 14.4 | 80.2 | 91.3 | 87.4 |
| THA-015 | Pv | CF11 + Culture | HiSeq | 100 | Yes | 2532 | 77.7 | 0.34 | - | 6.29 | 16.0 | 69.2 | 91.5 | 87.9 |
| THA-016 | Pv | CF11 + Culture | HiSeq | 100 | Yes | 3220 | 31.6 | 0.17 | - | 60.8 | 7.54 | 35.7 | 90.5 | 85.7 |
| THA-017 | Pv | CF11 + Culture | HiSeq | 100 | Yes | 1654 | 74.2 | 0.27 | - | 8.35 | 17.4 | 43.2 | 90.2 | 86.3 |
| THA-018 | Pv | CF11 + Culture | HiSeq | 100 | Yes | 2613 | 85.2 | 0.27 | - | 2.04 | 12.8 | 78.7 | 90.6 | 87.3 |
| THA-019 | Pv | CF11 + Culture | HiSeq | 100 | Yes | 2908 | 79.6 | 0.27 | - | 2.23 | 18.2 | 81.5 | 92.6 | 89.1 |
| THA-020 | Pv | CF11 + Culture | HiSeq | 100 | Yes | 3220 | 89.1 | 0.29 | - | 0.18 | 10.7 | 102 | 91.9 | 87.5 |
| DRW-001 | Pv | CF11 Only | GAII | 76 | No | 6911 | 24.0 | 0.18 | - | 64.6 | 11.4 | 54.3 | 90.7 | 85.9 |
| DRW-001 | Pv | CF11 + Culture | GAII | 76 | No | 5473 | 61.4 | 0.41 | - | 25.3 | 13.4 | 116 | 90.8 | 88.3 |
| DRW-002 | Pv | CF11 + Culture* | HiSeq | 75 | Yes | 4993 | 11.0 | 0.18 | - | 83.2 | 5.79 | 17.8 | 90.4 | 45.6 |
| DRW-003 | Pv | CF11 Only | HiSeq | 75 | Yes | 1840 | 20.4 | 0.15 | - | 71.8 | 7.83 | 12.6 | 91.5 | 9.65 |
| DRW-003 | Pv | CF11 + Culture | HiSeq | 75 | Yes | 1973 | 70.7 | 0.30 | - | 13.4 | 15.9 | 47.4 | 92.1 | 86.8 |
| DRW-004 | Pv + Pf | CF11 + Culture | HiSeq | 76 | No | 1829 | 8.6 | 0.25 | 38.1 | 47.7 | 5.60 | 5.38 | 87.8 | 0.32 |

Pv=*P. vivax*; Pf=*P. falciparum*; ^a^ Multiplexes comprise 12 samples. ^b^ Quantitative real-time PCR estimate; ^c^ Reads overlapping *P. vivax* and *P. falciparum*. ^d^ Expected sequence depth at each position in the *P. vivax* genome – calculated as total number of bases sequenced divided by number of bases in the genome (based on Sal-1 reference genome size ~27Mb). **^e^** Percentage Sal-1 reference genome covered at given read depth. *Poor maturation during culture.
