# Supplementary material for: Effective Preparation of Plasmodium vivax Field Isolates for High-Throughput Whole Genome Sequencing
Source: PLoS One. 2013 Jan 4;8(1):e53160. doi: 10.1371/journal.pone.0053160 (PMC3537768; doi:10.1371/journal.pone.0053160)
Supplement: Table S2 — Clinical and Laboratory Sample Properties. *Poor maturation during short-term culture. (DOCX) [file pone.0053160.s003.docx]

**Table S2: Clinical and Laboratory Sample Properties**

| **Origin** | **Sample** | **Processing** | **Parasite density (**µ**l^-1^)** | **% Rings pre-culture** | **% Schizonts post-culture** | **Duration culture (Hrs)** | **% Human** | **P. vivax yield (ng** µ**l^-1^ RBCs)** | **Total DNA (ng** µ**l^-1^ RBCs)** |
| --- | --- | --- | --- | --- | --- | --- | --- | --- | --- |
| **Travellers** | DRW-001 | Unprocessed | 84403 | 94 | - | - | 91.9 | 3.93 | 40.0 |
|  | DRW-001 | CF11 | 84403 | - | - | - | 24.3 | 1.91 | 6.55 |
|  | DRW-001 | CF11 + Culture | 84403 | - | 47 | 43 | 6.29 | 3.82 | 6.22 |
|  | DRW-002 | Unprocessed | 6000 | 7 | - | - | 95.2 | 1.29 | 13.6 |
|  | DRW-002 | CF11 | 6000 | - | - | - | 60.6 | 1.71 | 12.1 |
|  | DRW-002 | CF11 + Culture* | 6000 | - | 12 | 41 | 56.5 | 0.57 | 3.79 |
|  | DRW-003 | Unprocessed | 2600 | 88 | - | - | 99.4 | 0.36 | 3.94 |
|  | DRW-003 | CF11 | 2600 | - | - | - | 54.6 | 0.24 | 1.55 |
|  | DRW-003 | CF11 + Culture | 2600 | - | 53 | 39 | 38.1 | 2.00 | 9.62 |
|  |  |  |  |  |  |  |  |  |  |
| **Indonesia** | IND-001 | CF11 | 34967 | 93 | - | - | 12.7 | 0.51 | 1.16 |
|  | IND-001 | CF11 + Culture | 34967 | - | 80 | 46 | 3.00 | 12.2 | 15.9 |
|  | IND-002 | CF11 | 7084 | 92 | - | - | 41.9 | 0.01 | 0.05 |
|  | IND-002 | CF11 + Culture | 7084 | - | 45 | 46 | 3.11 | 5.84 | 7.66 |
|  | IND-003 | CF11 | 9719 | 95 | - | - | 68.6 | 0.01 | 0.08 |
|  | IND-003 | CF11 + Culture | 9719 | - | 32 | 50 | 10.0 | 11.6 | 23.2 |
|  | IND-004 | CF11 | 10256 | 100 | - | - | 25.9 | 0.04 | 0.14 |
|  | IND-004 | CF11 + Culture | 10256 | - | 54 | 48 | 8.93 | 5.33 | 10.1 |
|  | IND-005 | CF11 | 27883 | 99 | - | - | 44.6 | 1.39 | 7.59 |
|  | IND-005 | CF11 + Culture | 27883 | - | 53 | 48 | 8.19 | 14.4 | 26.2 |
|  | IND-006 | CF11 | 45844 | 97 | - | - | 42.9 | 0.25 | 1.32 |
|  | IND-006 | CF11 + Culture | 45844 | - | 20 | 53 | 23.0 | 2.34 | 7.72 |
|  | IND-007 | CF11 | 14771 | 96 | - | - | 18.6 | 0.11 | 0.31 |
|  | IND-007 | CF11 + Culture | 14771 | - | 35 | 48 | 4.24 | 3.47 | 4.94 |
|  | IND-008 | CF11 | 24680 | 97 | - | - | 1.58 | 0.18 | 0.21 |
|  | IND-008 | CF11 + Culture | 24680 | - | 46 | 48 | 0.20 | 6.56 | 6.69 |
|  |  |  |  |  |  |  |  |  |  |
|  | IND-009 | CF11 + Culture | 8213 | 95 | 58 | 43 | 3.21 | 9.39 | 12.4 |
|  | IND-010 | CF11 + Culture | 42604 | 95 | 41 | 50 | 3.57 | 4.61 | 6.26 |
|  | IND-011 | CF11 + Culture | 99475 | 94 | 50 | 48 | 4.31 | 5.22 | 7.47 |
|  | IND-012 | CF11 + Culture | 45844 | 96 | 36 | 44 | 0.83 | 6.98 | 7.56 |
|  | IND-013 | CF11 + Culture | 12434 | 95 | 20 | 48 | 17.43 | 0.56 | 1.54 |
|  | IND-014 | CF11 + Culture | 16956 | 80 | 24 | 47 | 8.9 | 1.17 | 2.21 |
|  | IND-015 | CF11 + Culture | 18700 | 97 | 42 | 45 | 8.62 | 2.52 | 4.69 |
|  |  |  |  |  |  |  |  |  |  |
| **Thailand** | THA-001 | CF11 + Culture | 37178 | 100 | 80 | 42 | 1.8 | 3.55 | 4.19 |
|  | THA-002 | CF11 + Culture | 32530 | 97 | 64 | 45 | 0.4 | 6.61 | 6.87 |
|  | THA-003 | CF11 + Culture | 41825 | 100 | 48 | 43 | 2.4 | 0.76 | 0.94 |
|  | THA-004 | CF11 + Culture | 41825 | 97 | 51 | 43 | 2.6 | 1.62 | 2.04 |
|  | THA-005 | CF11 + Culture | 35530 | 82 | 56 | 43 | 2.7 | 0.68 | 0.86 |
|  | THA-006 | CF11 + Culture | 18589 | 94 | 80 | 41 | 2.7 | 3.12 | 3.96 |
|  | THA-007 | CF11 + Culture | 7436 | 100 | 78 | 41 | 0.5 | 4.78 | 5.02 |
|  | THA-008 | CF11 + Culture | 9294 | 82 | 66 | 42 | 7.1 | 1.82 | 3.11 |
|  | THA-009 | CF11 + Culture | 13942 | 88 | 42 | 40 | 19.7 | 1.43 | 4.25 |
|  | THA-010 | CF11 + Culture | 37178 | 99 | 88 | 44 | 1.5 | 5.46 | 6.28 |
|  | THA-011 | CF11 + Culture | 13942 | 92 | 74 | 44 | 1.6 | 3.49 | 4.05 |
|  | THA-012 | CF11 + Culture | 18589 | 85 | 71 | 42 | 7.6 | 2.62 | 4.61 |
|  | THA-013 | CF11 + Culture | 9294 | 90 | 43 | 45 | 20.5 | 1.00 | 3.05 |
|  | THA-014 | CF11 + Culture | 13942 | 94 | 74 | 40 | 4.1 | 2.34 | 3.30 |
|  | THA-015 | CF11 + Culture | 27883 | 90 | 57 | 43 | 3.6 | 3.07 | 4.18 |
|  | THA-016 | CF11 + Culture | 37178 | 84 | 27 | 43 | 53.4 | 0.64 | 4.06 |
|  | THA-017 | CF11 + Culture | 9294 | 87 | 73 | 42 | 4.4 | 1.70 | 2.45 |
|  | THA-018 | CF11 + Culture | 41825 | 83 | 62 | 41 | 1.3 | 4.79 | 5.41 |
|  | THA-019 | CF11 + Culture | 18589 | 96 | 43 | 46 | 2.3 | 1.01 | 1.24 |
|  | THA-020 | CF11 + Culture | 27883 | 88 | 60 | 42 | 0.4 | - | - |

*Poor maturation during short-term culture.
