# Supplementary material for: Effective Preparation of Plasmodium vivax Field Isolates for High-Throughput Whole Genome Sequencing
Source: PLoS One. 2013 Jan 4;8(1):e53160. doi: 10.1371/journal.pone.0053160 (PMC3537768; doi:10.1371/journal.pone.0053160)
Supplement: Table S3 — European Nucleotide Archive Sample Accession Numbers. For ethics purposes, all samples were subject to human read alignment filtration prior to submission to the ENA. (DOCX) [file pone.0053160.s004.docx]

**Table S3: European Nucleotide Archive Sample Accession Numbers**

| Sample Origin | ENA Sample Accession Number |
| --- | --- |
| Thailand | ERS055892 |
| Thailand | ERS055878 |
| Thailand | ERS055895 |
| Thailand | ERS055889 |
| Thailand | ERS055896 |
| Thailand | ERS055885 |
| Thailand | ERS055881 |
| Thailand | ERS055887 |
| Thailand | ERS055888 |
| Thailand | ERS055877 |
| Thailand | ERS055882 |
| Thailand | ERS055884 |
| Thailand | ERS055893 |
| Thailand | ERS055886 |
| Thailand | ERS055883 |
| Thailand | ERS055891 |
| Thailand | ERS055890 |
| Thailand | ERS055880 |
| Thailand | ERS055894 |
| Thailand | ERS055879 |
| Darwin (traveller) | ERS017709 |
| Darwin (traveller) | ERS017708 |
| Darwin (traveller) | ERS040115 |
| Darwin (traveller) | ERS040112 |
| Darwin (traveller) | ERS040113 |
| Darwin (traveller) | ERS012057 |

For ethics purposes, all samples were subject to human read alignment filtration prior to submission to the ENA.
